# Supplementary material for: Activation of the urotensin-II receptor by remdesivir induces cardiomyocyte dysfunction
Source: Commun Biol. 2023 May 12;6:511. doi: 10.1038/s42003-023-04888-x (PMC10175918; doi:10.1038/s42003-023-04888-x)

Supplementary Information

Supplementary Figure 1. Activation of urotensin-II receptor (UTS2R) by remdesivir.

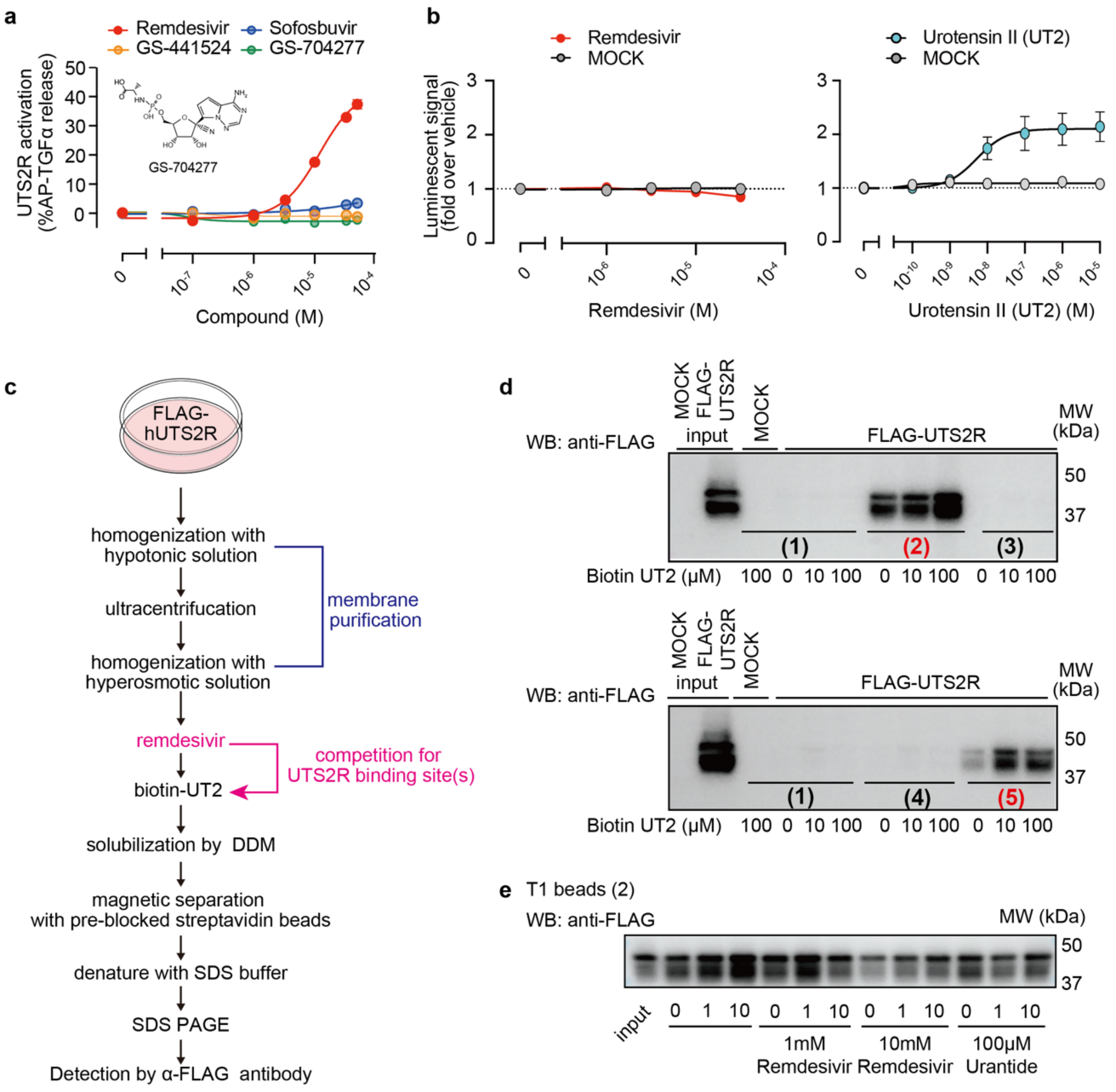

(a) Remdesivir-related compounds-mediated TGFα-shedding response curves for UTS2R. Data are shown as means ± SEM (n = 3). **(b)** (left) Remdesivir- or (right) urotensin-II (UT2)-mediated β-arrestin 2 recruitment assay for UTS2R. Data are shown

as means ± SEM (n = 3). **(c)** Experimental procedures of competitive binding assay. **(d)** Comparison of streptavidin magnetic beads. (1) Streptavidin magnetic beads (NEB, hydrophilic), (2) Dynabeads MyOne Streptavidin T1 (hydrophobic), (3) Dynabeads MyOne Streptavidin C1 (hydrophilic), (4) Dynabeads M-270 Streptavidin (hydrophilic), (5) Dynabeads M-280, Streptavidin (hydrophobic). Hydrophobic beads (2) or (5) served the aim for competitive binding assay. **(e)** UTS2R-bound biotin-UT2 detection using T1 beads (2). Membrane fraction of HEK293 cells expressing FLAG-UTS2R (input) was incubated with biotin-UT2 in the presence or absence of remdesivir or urantide. FLAG-UTS2R was then pulled down by streptavidin-coated T1 magnetic beads, and the amount of FLAG-UTS2R was detected by western blot.

Supplementary Figure 2. Effects of mutations on UTS2R activation.

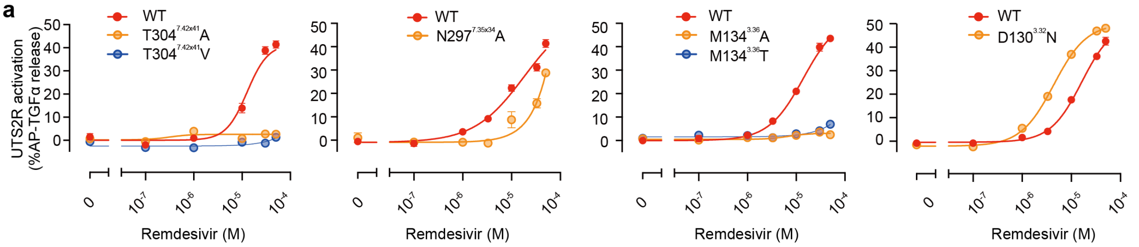

hUTS2R	WT	T304$^{7.42x41}$A	T304$^{7.42x41}$V	N297$^{7.35x34}$A	M134$^{3.36}$A	M134$^{3.36}$T	D130$^{3.32}$N
Remdesivir ⊿pEC_{50} cut off value: -1	0	< -1	< -1	-0.91 ± 0.086	< -1	< -1	0.53 ± 0.019

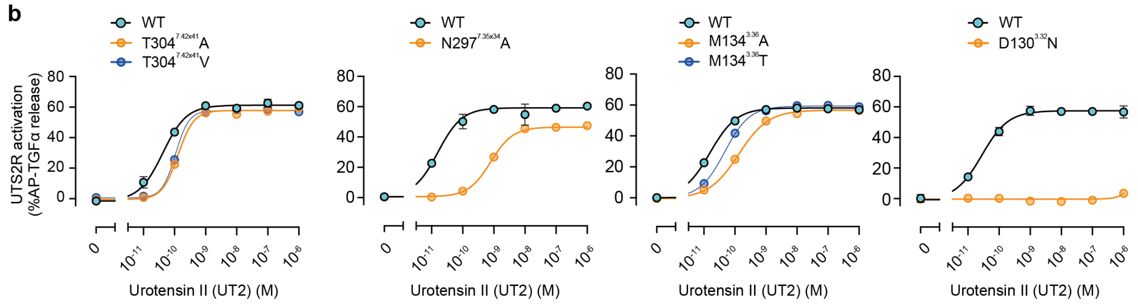

hUTS2R	WT	T304$^{7.42x41}$A	T304$^{7.42x41}$V	N297$^{7.35x34}$A	M134$^{3.36}$A	M134$^{3.36}$T	D130$^{3.32}$N
UT2 ⊿pEC_{50} cut off value: -1	0	-0.67 ± 0.091	-0.68 ± 0.16	< -1	-0.80 ± 0.026	-0.43 ± 0.13	< -1

(a, b) Remdesivir (a)- and UT2 (b)-mediated TGFα-shedding response curves for UTS2R WT and indicated mutants.⊿pEC_{50} value = pEC_{50} mutant - pEC_{50} WT. The ⊿pEC_{50} cutoff value was set to -1. EC_{50} values were determined by the TGFα-shedding assay. Data are shown as means ± SEM (n = 3).

Supplementary Figure 3. Intracellular signaling evoked by remdesivir-mediated UTS2R activation.

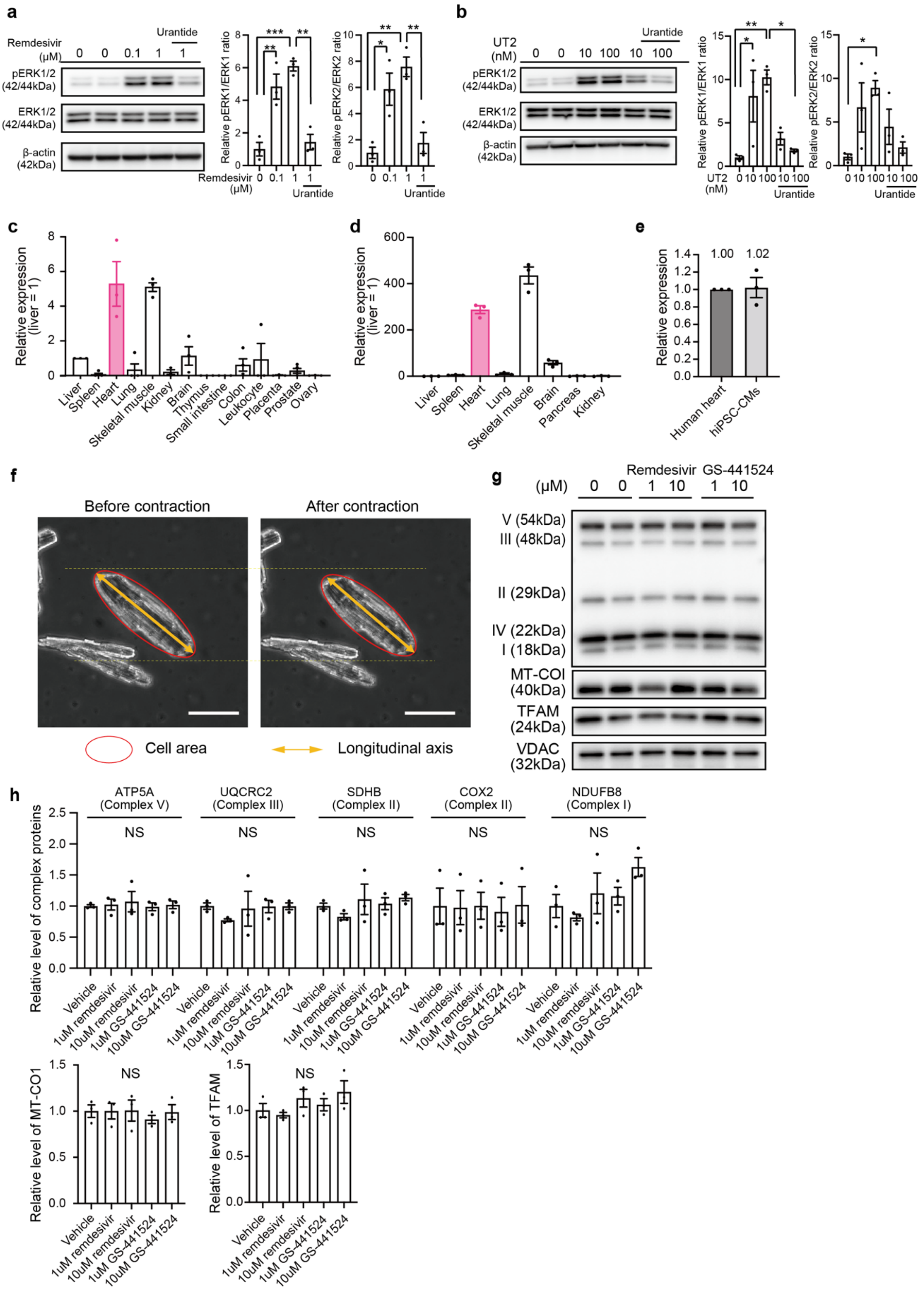

a
Remdesivir (μM)
0 0 0.1 1 1
Urantide
pERK1/2 (42/44kDa)
ERK1/2 (42/44kDa)
β-actin (42kDa)
Relative pERK1/ERK1 ratio
Relative pERK2/ERK2 ratio
***
**
*
Remdesivir (μM)
Urantide
b
UT2 (nM)
0 0 10 100 10 100
Urantide
pERK1/2 (42/44kDa)
ERK1/2 (42/44kDa)
β-actin (42kDa)
Relative pERK1/ERK1 ratio
Relative pERK2/ERK2 ratio
UT2 (nM)
0 10 100 10 100
Urantide
c
Relative expression (liver = 1)
Liver
Spleen
Heart
Lung
Skeletal muscle
Kidney
Brain
Thymus
Small intestine
Colon
Leukocyte
Placenta
Prostate
Ovary
d
Relative expression (liver = 1)
Liver
Spleen
Heart
Lung
Skeletal muscle
Brain
Pancreas
Kidney
e
Relative expression
1.00
1.02
Human heart
hiPSC-CMs
f
Before contraction
After contraction
Cell area
Longitudinal axis
g
(μM)
0 0
Remdesivir
1 10
GS-441524
1 10
V (54kDa)
III (48kDa)
II (29kDa)
IV (22kDa)
I (18kDa)
MT-COI (40kDa)
TFAM (24kDa)
VDAC (32kDa)
h
Relative level of complex proteins
ATP5A (Complex V)
UQCRC2 (Complex III)
SDHB (Complex II)
COX2 (Complex II)
NDUFB8 (Complex I)
NS
Vehicle
1uM remdesivir
10uM remdesivir
1uM GS-441524
10uM GS-441524
Relative level of MT-CO1
Relative level of TFAM
NS

(a) Serum-starved HEK293 cells overexpressing UTS2R were stimulated with the indicated concentrations of remdesivir for 72 h with or without urantide, a UTS2R antagonist, and the lysates subjected to western blotting analysis. ERK1 and ERK2 activation ratio (pERK1/ERK1 and pERK2/ERK2) were calculated with data normalized to the vehicle. * $p < 0.05$, ** $p < 0.01$, *** $p < 0.001$ by Tukey's multiple comparisons test. Data are shown as means ± SEM (n = 3). **(b)** Serum-starved HEK293 cells overexpressing UTS2R were stimulated with the indicated concentrations of UT2 for 5 min with or without urantide, a UTS2R antagonist, and the lysates subjected to western blotting analysis. ERK1 and ERK2 activation ratio were calculated with data normalized to the vehicle. * $p < 0.05$, ** $p < 0.01$ by Tukey's multiple comparisons test. Data are shown as means ± SEM (n = 3). **(c)** Relative expression levels of UTS2R in normal human tissues were assessed by qPCR using the Human Multiple Tissue cDNA (MTC) panel. Data are shown as means ± SEM (n = 3). **(d)** Relative expression levels of Uts2r in adult mouse tissues as assessed by qPCR. Data are shown as means ± SEM (n = 3). **(e)** Comparison of relative gene expression levels of UTS2R between human heart (using MTC panel) and human-induced pluripotent stem cell-derived cardiomyocytes (hiPSC-CMs). Data are shown as means ± SEM (n = 3). **(f)** Evaluation of adult cardiomyocyte contractile activity. Percentile change in cell area was calculated by dividing the cell area before contraction

by that after contraction. Ratio of cardiomyocyte shortening was calculated by dividing the longitudinal axis before contraction by that after contraction. Scale bars = 50 µm. **(g, h)** Expression levels of mitochondrial respiratory complex proteins in cells treated with remdesivir or GS-441524. HEK293 cells overexpressing UTS2R were stimulated with the indicated concentrations of remdesivir or GS-4415424 for 48 h. For each cell lysate sample, 10 µg of proteins were subjected to SDS-PAGE and western blotting. Each cropped image was taken from a separate blot. Mitochondrial respiratory complex proteins, namely MTCO1, TFAM, and VDAC were detected on separate membranes, respectively. The intensity of respiratory complex proteins, MTCO1, and TFAM were normalized to VDAC (single VDAC control with the same protein loaded (10 µg per lane, not run on each blot) and calculated with data normalized to the vehicle by Tukey's multiple comparisons test. Data are shown as means ± SEM (n = 3).

Supplementary Figure 4. Effects of SNV on remdesivir-mediated UTS2R activation.

a

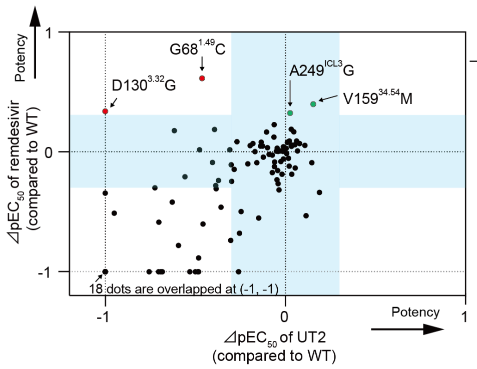

b

Position (GRCh38.p13)	Alleles	Amino acid	Position (Amino acid)	rs#	Frequency
chr17:82374526	G>T	G (Gly) > C (Cys)	68	N/A	0.00007 (gnomAD AMR)
chr17:82374713	A>G	D (Asp) > G (Gly)	130	rs1318518750	0.00019 (gnomAD EAS)
chr17:82374799	G>A	V (Val) > M (Met)	159	rs757940874	0.00005 (gnomAD AFR)
chr17:82375070	C>G	A (Ala) > G (Gly)	249	rs972689441	0.00002 (gnomAD AFR)

(a) Scatter plot of ⊿pEC_{50} of remdesivir vs. ⊿pEC_{50} of UT2 for 110 missense human SNVs. Light blue bands represent the range of -0.3< ⊿pEC_{50} <0.3, which corresponds to the range of >0.5-fold and <2-fold change in EC_{50} compared to the WT receptor. Mutations that increase the receptor sensitivity toward remdesivir (⊿pEC_{50} >0.3) but not toward the UT2 (⊿pEC_{50} <0) are marked in red. Mutations that increase the receptor sensitivity toward both remdesivir and UT2 (⊿pEC_{50} >0.3 for remdesivir, 0< ⊿pEC_{50} <0.3 for UT2) are marked in green. All experiments were performed in triplicate, and the data are expressed as the means. **(b)** Allele frequencies of the four gain-of-function mutations ($G68^{1.49}C$, $D130^{3.32}G$, $V159^{34.54}M$, and $A249^{ICL3}G$). Data are modified from the 14KJPN Genome Reference Panel (https://jmorp.megabank.tohoku.ac.jp/202112/).

Supplementary Figure 5. Original Western Blots

The original blots for Figures 1d; 3a; 4c; 4d; Supplementary Figures 1d; 1e; 3a; 3b; 3g.

Fig 1d

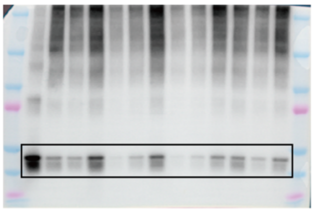

Fig 3a
pERK

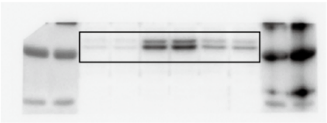

Fig 3a
ERK

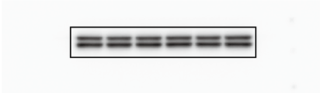

Fig 3a
β-actin

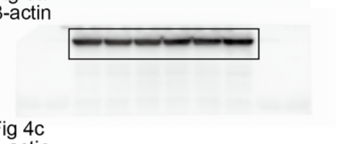

Fig 4c
pERK

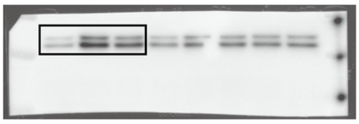

Fig 4c
ERK

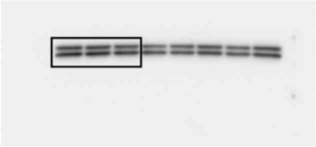

Fig 4c
β-actin

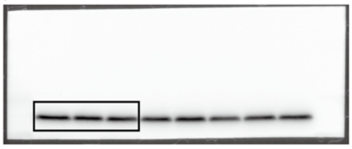

Fig 4d
pAkt

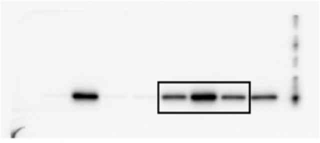

Fig 4d
Akt

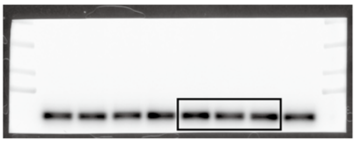

Fig 4d
β-actin

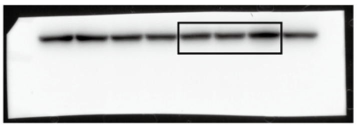

Supp Fig 1d
beads (1), (2), (3)

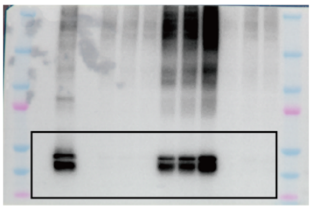

beads (1), (4), (5)

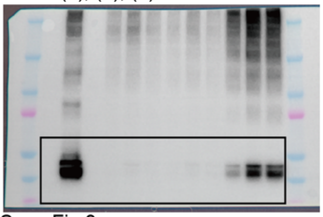

Supp Fig 1e

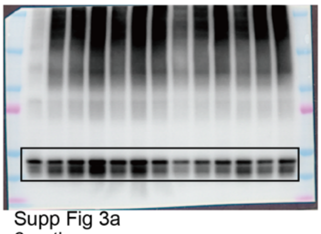

Supp Fig 3a
pERK

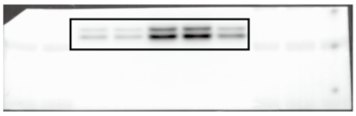

Supp Fig 3a
ERK

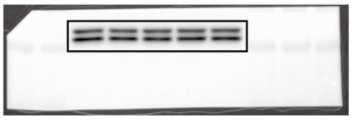

Supp Fig 3a
β-actin

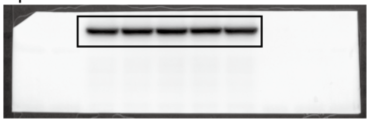

Supp Fig 3b
pERK

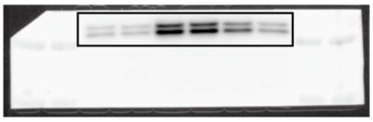

Supp Fig 3b
ERK

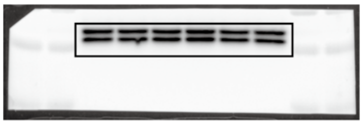

Supp Fig 3b
β-actin

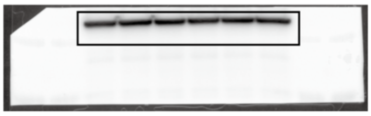

Supp Fig 3g
OXPHOS

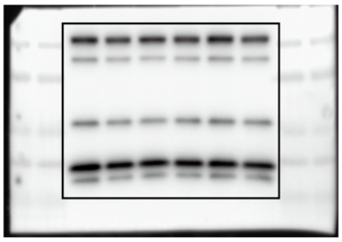

Supp Fig 3g
MT-COI

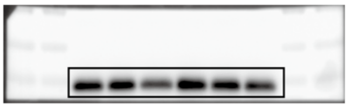

Supp Fig 3g
TFAM

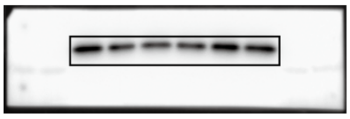

Supp Fig 3g
VDAC

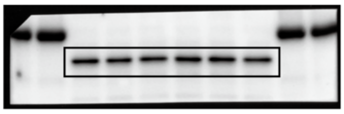

Supplement: Supplementary file 1 — Supplementary Information [file 42003_2023_4888_MOESM1_ESM.pdf]
