# Supplementary material for: Activation of the urotensin-II receptor by remdesivir induces cardiomyocyte dysfunction
Source: Commun Biol. 2023 May 12;6:511. doi: 10.1038/s42003-023-04888-x (PMC10175918; doi:10.1038/s42003-023-04888-x)
Supplement: Supplementary file 2 — Description of Additional Supplementary Files [file 42003_2023_4888_MOESM2_ESM.docx]

**Description of Additional Supplementary Files**

**File name:** Supplementary Data 1

**Description:** The source data behind the graphs in the paper

**File name:** Supplementary Data 2

**Description:** Effects of missense SNVs in UTS2R gene on receptor activation

**File name:** Supplementary Data 3

**Description:** Detailed sample information of pooled human cDNA

**File name:** Supplementary Data 3

**Description:** Sequences of primers used for quantitative PCR
