# Supplementary material for: Activation of the urotensin-II receptor by remdesivir induces cardiomyocyte dysfunction
Source: Commun Biol. 2023 May 12;6:511. doi: 10.1038/s42003-023-04888-x (PMC10175918; doi:10.1038/s42003-023-04888-x)
Supplement: Supplementary file 7 — Reporting Summary [file 42003_2023_4888_MOESM7_ESM.pdf]

nature portfolio

Corresponding author(s): Fan-Yan Wei

Last updated by author(s): 2023/4/13

Reporting Summary

Nature Portfolio wishes to improve the reproducibility of the work that we publish. This form provides structure for consistency and transparency in reporting. For further information on Nature Portfolio policies, see our Editorial Policies and the Editorial Policy Checklist.

Statistics

For all statistical analyses, confirm that the following items are present in the figure legend, table legend, main text, or Methods section.

n/a	Confirmed	
☐	☒	The exact sample size (*n*) for each experimental group/condition, given as a discrete number and unit of measurement
☐	☒	A statement on whether measurements were taken from distinct samples or whether the same sample was measured repeatedly
☐	☒	The statistical test(s) used AND whether they are one- or two-sided<br>*Only common tests should be described solely by name; describe more complex techniques in the Methods section.*
☒	☐	A description of all covariates tested
☐	☒	A description of any assumptions or corrections, such as tests of normality and adjustment for multiple comparisons
☐	☒	A full description of the statistical parameters including central tendency (e.g. means) or other basic estimates (e.g. regression coefficient) AND variation (e.g. standard deviation) or associated estimates of uncertainty (e.g. confidence intervals)
☐	☒	For null hypothesis testing, the test statistic (e.g. *F*, *t*, *r*) with confidence intervals, effect sizes, degrees of freedom and *P* value noted<br>*Give P values as exact values whenever suitable.*
☒	☐	For Bayesian analysis, information on the choice of priors and Markov chain Monte Carlo settings
☒	☐	For hierarchical and complex designs, identification of the appropriate level for tests and full reporting of outcomes
☒	☐	Estimates of effect sizes (e.g. Cohen's *d*, Pearson's *r*), indicating how they were calculated

Our web collection on statistics for biologists contains articles on many of the points above.

Software and code

Policy information about availability of computer code

Data collection	No software was used for data collection.
Data analysis	GraphPad Prism (Version 9.4.1) and Microsoft excel (Version 16.66.1) were used to calculate mean, standard error and P value. Fiji software was used to analyze imaging data.

For manuscripts utilizing custom algorithms or software that are central to the research but not yet described in published literature, software must be made available to editors and reviewers. We strongly encourage code deposition in a community repository (e.g. GitHub). See the Nature Portfolio guidelines for submitting code & software for further information.

Data

Policy information about availability of data

All manuscripts must include a data availability statement. This statement should provide the following information, where applicable:

- Accession codes, unique identifiers, or web links for publicly available datasets
- A description of any restrictions on data availability
- For clinical datasets or third party data, please ensure that the statement adheres to our policy

All data are available in the main text or the supplementary materials.

Research involving human participants, their data, or biological material

Policy information about studies with human participants or human data. See also policy information about sex, gender (identity/presentation), and sexual orientation and race, ethnicity and racism.

Reporting on sex and gender	not applicable
Reporting on race, ethnicity, or other socially relevant groupings	not applicable
Population characteristics	not applicable
Recruitment	not applicable
Ethics oversight	not applicable

Note that full information on the approval of the study protocol must also be provided in the manuscript.

Field-specific reporting

Please select the one below that is the best fit for your research. If you are not sure, read the appropriate sections before making your selection.

☒ Life sciences ☐ Behavioural & social sciences ☐ Ecological, evolutionary & environmental sciences

For a reference copy of the document with all sections, see nature.com/documents/nr-reporting-summary-flat.pdf

Life sciences study design

All studies must disclose on these points even when the disclosure is negative.

Sample size	Sample size was chosen based on the calculation by G*Power3.1.9.2 software, or on previous experience by our group on similar experiments (PMID 33472058)
Data exclusions	No data were excluded.
Replication	Replicate experiments were successful.
Randomization	Laboratory animals were randomly assigned to experimental groups. The order of treatment administration was also randomized. All cell samples were randomly selected for experiments.
Blinding	Blinding was not relevant to this study because no bias could be made in the experiments performed.

Reporting for specific materials, systems and methods

We require information from authors about some types of materials, experimental systems and methods used in many studies. Here, indicate whether each material, system or method listed is relevant to your study. If you are not sure if a list item applies to your research, read the appropriate section before selecting a response.

Materials & experimental systems

n/a	Involved in the study	
☐	☒	Antibodies
☐	☒	Eukaryotic cell lines
☒	☐	Palaeontology and archaeology
☐	☒	Animals and other organisms
☒	☐	Clinical data
☒	☐	Dual use research of concern
☒	☐	Plants

Methods

n/a	Involved in the study	
☒	☐	ChIP-seq
☒	☐	Flow cytometry
☒	☐	MRI-based neuroimaging

Antibodies

Antibodies used	nti-β-Actin mAb, Code No.M177-3, MBL<br>Phospho-p44/42 MAPK (Erk1/2) (Thr202/Tyr204) Rabbit mAb, #9101, Cell Signaling<br>p44/42 MAPK (Erk1/2) (137F5) Rabbit mAb #4695, Cell Signaling

	Phospho-Akt (Ser473) (D9E) XP® Rabbit mAb #4060, Cell Signaling<br>Akt (pan) (C67E7) Rabbit mAb #4691, Cell Signaling<br>Anti-MTCO1 antibody [1D6E1A8], ab14705, abcam<br>TFAM (D5C8) Rabbit mAb #8076, Cell Signaling<br>Total OXPHOS Human WB Antibody Cocktail, ab110411, abcam<br>Anti-FLAG antibody (M2), #F3165, Sigma
Validation	All antibodies are commercially available and were commercially validated.

Eukaryotic cell lines

Policy information about cell lines and Sex and Gender in Research

Cell line source(s)	For the GPCR assay, we used HEK293 cells obtained from ATCC.
Authentication	The cell line used was not authenticated.
Mycoplasma contamination	The cell lines was tested negative for mycoplasma contamination.
Commonly misidentified lines<br>(See ICLAC register)	No commonly misidentified cell lines were used.

Animals and other research organisms

Policy information about studies involving animals; ARRIVE guidelines recommended for reporting animal research, and Sex and Gender in Research

Laboratory animals	Sprague-Dawley rat pups (male and female) on postnatal day 1-3 were purchased from Japan SLC, Inc.<br>C57BL/6J mice for the isolation of adult mouse cardiomyocytes were purchased from Japan SLC Inc.
Wild animals	No wild animals were used.
Reporting on sex	not applicable
Field-collected samples	No field-collected samples were used.
Ethics oversight	Animal studies were reviewed and approved by the ethics committees at the Tohoku University and Kyushu University and carried out in accordance with their guidelines.

Note that full information on the approval of the study protocol must also be provided in the manuscript.
